# GDF15 as a sentinel of metabolic integrity: a narrative review of stress signaling, multisystem pathophysiology, and therapeutic potential

**DOI:** 10.3389/fendo.2026.1885104

**Published:** 2026-09-16

**Authors:** Tao Nie, Hongmei Song, Xiaofang Shen, Liufeng Mao

**Affiliations:** 1 Medical College, Hubei University of Arts and Science, Xiangyang, Hubei, China; 2 The First Affiliated Hospital, Guangdong Pharmaceutical University, Guangzhou, China; 3 Neurological Research Institute of Integrated Traditional Chinese and Western Medicine, The First Affiliated Hospital, Guangdong Pharmaceutical University, Guangzhou, China

**Keywords:** cardiometabolic disease, GDF15, GFRAL, metabolic integrity, obesity

## Abstract

Growth differentiation factor 15 (GDF15) is a stress responsive cytokine belonging to the transforming growth factor-β (TGF-β) superfamily, serving as a sentinel of metabolic integrity. Its expression is induced by multiple convergent stress pathways, including the integrated stress response (ISR), endoplasmic reticulum (ER) stress, oxidative stress, inflammation, and hypoxia. GDF15 is secreted by a wide range of organs, such as adipose tissue, skeletal muscle, liver, heart, kidney, immune cells, and the central nervous system, with the relative contribution of each tissue varying across different physiological and pathological states. Through the hindbrain GFRAL-RET axis, GDF15 suppresses appetite and regulates energy balance, while also exerting peripheral effects on mitochondrial function, insulin sensitivity, and inflammatory responses. Transient GDF15 elevation in response to acute stressors supports adaptive metabolic resilience and tissue protection. In contrast, chronic and sustained elevation signals progressive metabolic dysfunction, organ impairment, and poor prognosis in conditions including obesity, metabolic dysfunction associated steatotic liver disease (MASLD), diabetes, and cardiovascular disease. Importantly, the biological impact of GDF15 is modified by factors such as age and metabolic context, which may shift the balance between adaptive and maladaptive outcomes and account for much of the predictive signal in clinical studies. Although GDF15 holds promise as a prognostic biomarker and a therapeutic target, its clinical translation is hindered by two major challenges: its dual protective and pathological roles, and an incomplete understanding of non-GFRAL signaling pathways. This narrative review summarizes the regulatory networks, tissue sources, and context-dependent functions of GDF15, with an emphasis on its roles in cardiometabolic homeostasis and the potential for future precision therapeutic strategies. We present the sentinel model as a working hypothesis that generates testable predictions, rather than as a proven biological mechanism. The acute-adaptive versus chronic-maladaptive distinction, while conceptually useful, requires validation through measurable parameters including concentration thresholds, temporal dynamics, and tissue specific contributions.

## 1 Introduction

Obesity and cardiometabolic disease are no longer adequately described as isolated abnormalities of body weight or circulating lipids (1, 2). Instead, they reflect progressive failure of metabolic integrity, characterized by chronic nutrient surplus, ectopic lipid deposition, mitochondrial dysfunction, ER stress, inflammatory activation and maladaptive inter-organ crosstalk (3–5). These perturbations impose a persistent burden on cellular quality control systems and on the capacity of organs to maintain homeostasis under energetic stress (6, 7).

Within this framework, circulating stress signals become especially informative. Among them, Growth differentiation factor 15 (GDF15) has emerged as one of the most biologically consequential. Its elevation across obesity, diabetes, metabolic dysfunction associated steatotic liver disease (MASLD), cardiovascular disease, frailty and cachexia suggests that it is not simply a marker of adiposity, but rather a readout of the degree to which metabolic homeostasis is threatened (8, 9). This interpretation is reinforced by the observation that GDF15 is induced by multiple stress response programs, including the ISR, mitochondrial stress and inflammatory stress (10, 11). It is important to note, however, that the evidence for GDF15 is heterogeneous: human studies are predominantly correlational and demonstrate associations with disease states and outcomes, whereas causal mechanisms have been primarily established through mouse genetic models and *in vitro* experiments. Throughout this review, we distinguish between these evidence types where the literature permits.

Interest in GDF15 accelerated significantly after the discovery that its anorexigenic effects are mediated by the brainstem receptor GDNF family receptor alpha-like (GFRAL), which signals through Ret proto-oncogene (RET) to suppress food intake (12–14). This finding established GDF15 as a bona fide endocrine regulator of appetite and body weight. Circulating GDF15 is elevated in a broad array of physiologically and pathologically distinct settings, including tissue injury, exercise, obesity, chronic kidney disease, cancer, heart failure and aging (15–19). The interpretive challenge is therefore not whether GDF15 is relevant, but how it should be interpreted. A biomarker that rises in both adaptive and maladaptive contexts must be embedded in a framework that distinguishes compensatory stress signaling from pathological disease burden. For GDF15, that framework is best captured by the concept of threatened metabolic integrity.

We propose that GDF15 should be understood as a context-dependent endocrine sentinel of metabolic integrity. This is presented as a working hypothesis that generates specific, testable predictions rather than as a proven conclusion. Specifically, the hypothesis predicts that the relationship between GDF15 concentration and clinical outcome should be nonlinear or threshold dependent, with low or transient elevation associated with adaptation and high or sustained elevation associated with pathology. Additional predictions are that the dominant tissue source of GDF15 should shift with disease stage and that therapeutic modulation of GDF15 should be stage dependent, with benefit in acute settings and potential harm in chronic settings. These predictions can be tested through longitudinal cohort studies with serial measurements, tissue specific intervention models, and stage stratified clinical trials. We acknowledge that the current literature does not yet provide definitive answers, and we highlight these as priorities for future investigation. In obesity, it may function as a compensatory brake on further nutrient intake. In MASLD and diabetes, it may reflect progressive hepatometabolic stress and disease severity. In cardiovascular disease, it may indicate declining physiological reserve and poor prognosis. This review integrates the molecular biology of GDF15, the stress pathways that regulate its expression, the tissue sources that shape its circulating pool, and the clinical contexts that determine its biological meaning (**Figure 1**).

**Figure 1:**
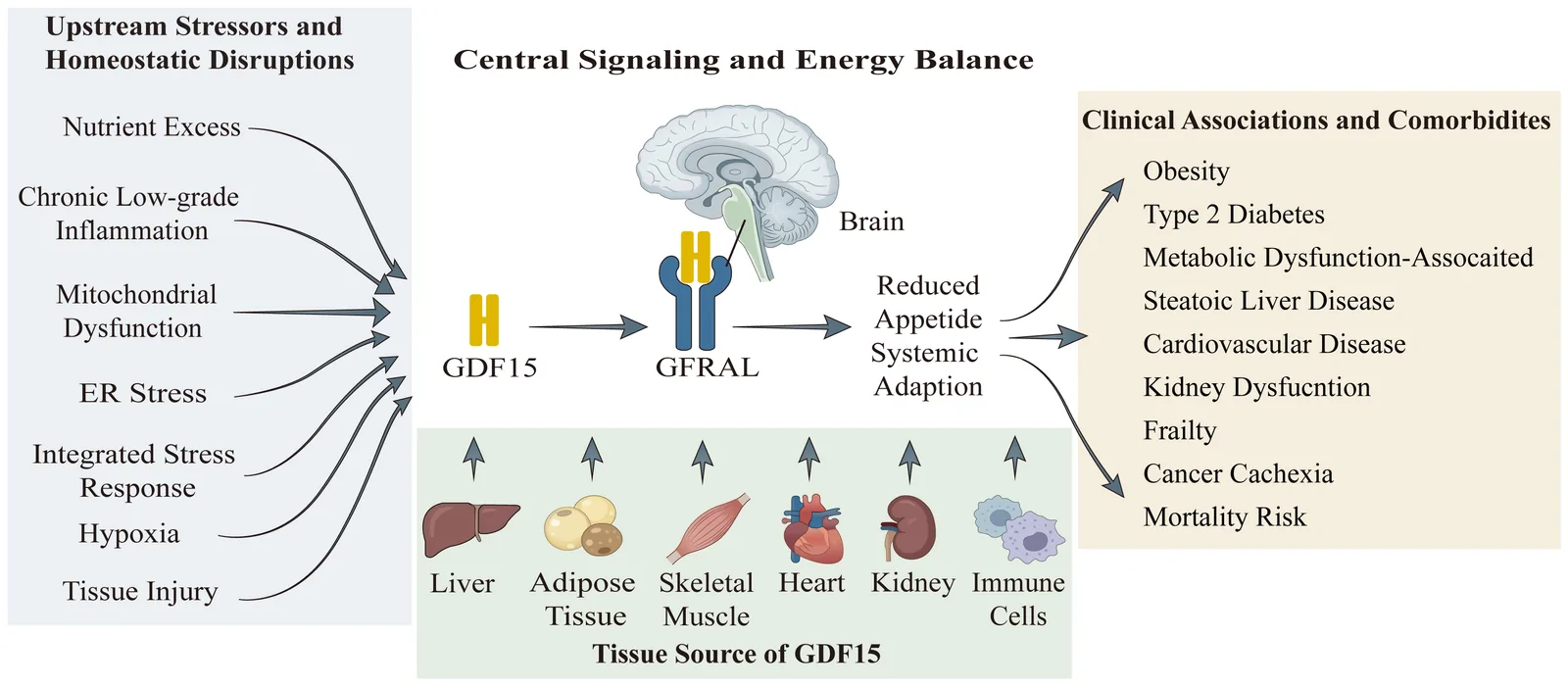
GDF15 as a sentinel of metabolic integrity. GDF15 functions as a context-dependent sentinel of metabolic integrity, integrating upstream stressors, multi-organ sources, central signaling, and clinical outcomes. Upstream stressors, including nutrient excess, chronic low-grade inflammation, mitochondrial dysfunction, ER stress (endoplasmic reticulum stress), ISR (integrated stress response), hypoxia, and tissue injury, converge to induce GDF15 expression across multiple organs (tissue sources, bottom). Once secreted, GDF15 acts through the hindbrain GFRAL-RET axis (GDNF family receptor alpha-like–Ret proto-oncogene) to suppress appetite and regulate energy balance, while also exerting peripheral effects on adipose tissue and skeletal muscle. In the context of threatened metabolic integrity, elevated GDF15 is associated with a broad spectrum of clinical associations and comorbidities, including obesity, type 2 diabetes, MASLD (metabolic dysfunction-associated steatotic liver disease), cardiovascular disease, kidney dysfunction, frailty, cancer cachexia, and mortality risk. This framework highlights that GDF15 functions not as a simple beneficial or harmful molecule, but as a context-dependent sentinel whose interpretation requires consideration of the nature, duration, and magnitude of upstream stress signals.

## 2 Molecular identity and core biology of GDF15

### 2.1 GDF15 as a divergent member of the TGF-β superfamily

GDF15 is a divergent member of the transforming growth factor-β (TGF-β) superfamily and has been described under several historical names, including macrophage inhibitory cytokine-1 (MIC-1) and non-steroidal anti-inflammatory drug-activated gene-1 (NAG-1) (20, 21). It is synthesized as a precursor protein that undergoes cleavage and is subsequently secreted into the circulation. Although the protein is widely expressed, basal levels remain low under healthy conditions, whereas expression rises sharply in response to stress, injury, and disease (22–25). This broad inducibility supports the role of GDF15 as a systemic stress sensor rather than a classical tissue restricted hormone. In this sense, it resembles other damage associated endocrine factors whose primary function is to report cellular strain to distal target organs.

### 2.2 GFRAL-RET signaling: the canonical pathway

A defining advance in GDF15 biology was the identification of GFRAL as its receptor. The anorectic and metabolic effects of GDF15 are mediated almost exclusively through GFRAL, which is expressed in a highly restricted population of excitatory neurons in the area postrema (AP) and nucleus tractus solitarius (NTS) of the brainstem (12, 26). GDF15 binds to GFRAL, which signals via the co-receptor RET, leading to activation of downstream pathways including ERK and AKT (27). This GDF15-GFRAL-RET axis represents a non-homeostatic emergency circuit that shapes feeding responses to stressful conditions (14). Activation of GFRAL^+^ neurons in the AP/NTS produces potent anorectic effects and conditioned taste aversion, linking pathophysiological states to the suppression of food intake. These neurons project extensively to the PBN and central amygdala, where they target CGRP-expressing neurons to mediate aversive responses (28). The AP/NTS is also the site of action for other appetite regulating hormones, and GDF15 signaling acts synergistically with leptin and GLP-1 pathways to enhance weight loss (29, 30).

It is important to note, however, that the role of the GDF15-GFRAL-RET axis in mediating the glycemic effects of metformin remains controversial. While Coll et al. demonstrated that metformin-induced improvements in glucose tolerance were independent of GDF15 (31), Day et al. reported that both the glucose lowering and insulin sensitizing effects of metformin were abolished in *Gdf15* null mice (32). In a subsequent study, Klein et al. found that metformin lowered body weight and improved glucose handling in diet induced obese mice independently of GDF15 and GFRAL, challenging the necessity of this pathway for metformin’s metabolic benefits (33). More recently, Zhang et al. provided evidence that acute activation of GFRAL in the area postrema directly contributes to glucose regulation independent of changes in food intake and body weight, suggesting that the GDF15-GFRAL axis may have weight independent effects on glucose homeostasis under certain conditions (34). These discrepancies may arise from differences in experimental protocols, including diet composition, metformin dosing and route of administration, treatment duration, genetic background of mouse strains, and environmental housing conditions (35). Collectively, these findings underscore that while GDF15-GFRAL signaling is clearly involved in metformin’s effects on energy balance, its contribution to glycemic control appears to be context-dependent and may not be universally required. Further studies are warranted to delineate the conditions under which this pathway contributes to metformin’s glucose lowering actions.

Furthermore, GDF15 acts directly on hypothalamic 4B cells to stimulate corticotropin-releasing factor (CRF) expression, indicating a role in activating the hypothalamic-pituitary-adrenal (HPA) axis in response to stressors such as genotoxic or ER toxins (36, 37). However, this observation derives from a single immortalized cell line and awaits confirmation in primary neurons or *in vivo* models. The receptor mediating this effect has not been identified (see Section 2.4).

However, the central GFRAL-RET axis likely represents only one facet of GDF15 biology. The restricted distribution of GFRAL suggests that the systemic consequences of GDF15 cannot be fully understood without considering upstream tissue specific induction and the broader network of downstream effects. Recent work has also highlighted context-dependent actions of GDF15 beyond appetite suppression, including its associations with energy expenditure, insulin sensitivity, and mitochondrial adaptation (38–41).

### 2.3 Distinguishing physiological from pathological expression

Under healthy conditions, GDF15 is expressed at relatively low levels but can be induced by adaptive stressors such as exercise and ketogenic diet (16, 42, 43). In disease states, its levels rise in obesity, diabetes, MASLD, cardiovascular disease, chronic kidney disease, and cancer (44). Thus, the same molecular signal may reflect either an adaptive response to transient stress or a maladaptive marker of chronic tissue injury (8). This duality is central to the interpretation of GDF15 levels. Persistent elevation in a chronically ill patient may signal ongoing disease burden, whereas transient elevation during exercise or metabolic adaptation may reflect physiological resilience. The biomarker value of GDF15, therefore, depends as much on temporal dynamics as on absolute concentration (45).

### 2.4 Beyond GFRAL: emerging evidence for non-canonical pathways

While the GFRAL-RET axis in the hindbrain mediates the anorexigenic effects of GDF15, accumulating evidence indicates that GDF15 exerts additional biological actions that are independent of this receptor. Given that GFRAL expression is largely restricted to the AP and NTS, the pleiotropic peripheral effects of GDF15, particularly on immune cells, cardiovascular tissues, and metabolism, cannot be fully explained by canonical GFRAL signaling.

Several lines of evidence support GFRAL-independent mechanisms. First, GDF15 directly modulates immune cell function through alternative receptors. In T cells, GDF15 interacts with CD48 to promote regulatory T cell generation and suppressive function (46). In macrophages and dendritic cells, GDF15 signals through TGF-β receptors I and II (TGFBR1/ALK5 and TGFBR2) to induce tolerogenic phenotypes and suppress inflammatory responses, effects that occur in the absence of detectable GFRAL (47). Second, GDF15 directly influences cell survival and proliferation through erb-b2 receptor tyrosine kinase 2 (ERBB2)/human epidermal growth factor receptor 2 (HER2). In pancreatic β-cells, GDF15 binds ERBB2 to reduce caspase-8 expression and protect against cytokine-induced apoptosis (48). In cervical and breast cancer cells, GDF15 interacts with ERBB2/HER2 to activate PI3K/AKT and MAPK/ERK pathways, promoting proliferation and therapy resistance (49, 50). Third, GDF15 has been shown to activate BMPR1A/BMPR2/p-SMAD1 signaling in trophoblast cells to enhance cell invasion and improve pregnancy outcomes (51).

The molecular identity of these non-canonical pathways is gradually being elucidated, with TGF-β receptors, ERBB2/HER2, CD48, and BMP receptors emerging as bona fide alternative receptors or signaling partners. Elucidating these mechanisms remains a high priority for the field, as it would enable tissue specific therapeutic targeting of GDF15’s protective or pathological actions without disrupting its central appetite regulating functions.

It is important to note, however, that a major caveat in the interpretation of non-canonical GDF15 signaling pathways is the potential for TGF-β contamination in recombinant GDF15 preparations. Olsen et al. demonstrated that commercially available mammalian cell-derived GDF15 batches contained variable amounts of TGF-β (ranging from 0 to 169.7 pg TGF-β per μg GDF15), and that SMAD2 activation originally attributed to GDF15 was in fact mediated by contaminating TGF-β (52). Given that TGF-β is biologically active in the femtomolar range, even trace contamination can confound results, particularly in studies using high concentrations of recombinant GDF15 (50–400 ng/mL) and cells expressing TGF-β receptors. This concern is especially relevant to the studies cited above that implicate TGFBR1/ALK5 and TGFBR2 as GDF15 receptors (47), as these are the canonical receptors for TGF-β itself. Thus, while non-GFRAL pathways may exist, the evidence for TGF-β receptor mediated GDF15 signaling should be interpreted with caution, and rigorous controls, including the use of TGF-β neutralizing antibodies and multiple independently purified protein batches, are essential for future studies in this area.

### 2.5 Ghrelin and LEAP2: the opposing orexigenic system in the AP/NTS

The AP/NTS integrates opposing peripheral signals to fine-tune energy balance. While GDF15 suppresses appetite via GFRAL-RET, this anorexigenic tone is functionally counterbalanced by the ghrelin system. Ghrelin, a 28-amino acid peptide acylated by ghrelin O-acyltransferase (GOAT), is produced mainly in the stomach and acts through its receptor GHSR (growth hormone secretagogue receptor), a Gq-coupled GPCR highly expressed in the AP and NTS as well as in the hypothalamus (53, 54). Hindbrain ghrelin administration potently stimulates food intake, placing GDF15 and ghrelin as opposing endocrine signals operating within the same neuroanatomical hub (55, 56).

Critically, GHSR exhibits ligand-independent constitutive activity, which contributes to basal hunger drive and modulates the sensitivity of the AP/NTS circuit to both orexigenic and anorexigenic inputs (57). This constitutive activity is inhibited by LEAP2 (Liver-Expressed Antimicrobial Peptide 2), an endogenous peptide that functions as both a competitive antagonist and an inverse agonist at GHSR (58, 59). LEAP2 levels are dynamically regulated by nutritional state, decreasing during fasting and rising postprandially, and inversely correlate with hunger sensations in humans, independently of ghrelin (60, 61). Beyond homeostatic feeding, LEAP2 also attenuates ghrelin-induced dopamine release in the mesolimbic reward system, implicating it in hedonic feeding (62).

The ghrelin-LEAP2-GHSR triad and the GDF15-GFRAL axis thus represent two functionally opposed, yet intimately connected, regulatory systems within the AP/NTS. Ghrelin signals energy deficit and promotes feeding, whereas GDF15 signals stress and suppresses appetite. In obesity, GDF15 rises as a compensatory response to nutrient excess, while ghrelin levels are paradoxically reduced and the ghrelin/LEAP2 ratio declines, reflecting complex homeostatic maladaptation (63). Therapeutically, GHSR antagonists and LEAP2 mimetics offer complementary strategies to GDF15-based approaches for appetite suppression, though their distinct physiological domains, homeostatic hunger versus stress induced satiety, warrant careful consideration (30, 64).

In summary, the AP/NTS does not simply receive a single anorexigenic input from GDF15; rather, it integrates opposing signals from the ghrelin-LEAP2-GHSR system. Dysregulation of this balance may underlie the persistent metabolic dysfunction observed in obesity and related cardiometabolic diseases.

## 3 Upstream regulation of GDF15: a convergent stress response network

### 3.1 Integrated stress response

The ISR is one of the most important regulatory pathways controlling GDF15 expression. Diverse insults, including amino acid deprivation, proteotoxic stress, mitochondrial dysfunction, and translational perturbation, converge on phosphorylation of eukaryotic translation initiation factor 2α (eIF2α), leading to selective translation of activating transcription factor 4 (ATF4). ATF4 subsequently induces downstream stress-responsive genes, including *Gdf15* (65, 66).

The ISR regulates GDF15 as a downstream stress induced secreted factor across multiple tissues, although the specific signaling routes are context-dependent. In adipocytes, ISR activation induces GDF15 through the direct action of ATF4 and CHOP (C/EBP Homologous Protein), linking eIF2α phosphorylation to transcriptional upregulation of the *Gdf15* gene. In renal injury and gut damage, the PKR-linked ISR is positively associated with *Gdf15* expression, and genetic loss of *Gdf15* exacerbates tissue injury, supporting a functional ISR-GDF15 axis during inflammatory and metabolic stress (36, 37). In the central nervous system, persistent ISR activation also drives GDF15 production. In models of vanishing white matter disease, GDF15 levels increase in stressed primary astrocytes and in retinal ganglion cells after optic nerve crush, whereas treatment with the eIF2B activator 2BAct suppresses both ISR signaling and GDF15 expression (38). Collectively, the ISR provides a unifying molecular bridge between cellular stress and the endocrine output of GDF15.

### 3.2 Endoplasmic reticulum stress

ER stress and the unfolded protein response (UPR) are tightly coupled to ISR activation and are prominently featured in obesity, MASLD, and insulin resistance. When the protein folding capacity of the ER is exceeded, protein kinase RNA-like endoplasmic reticulum kinase (PERK) mediated signaling contributes to translational reprogramming and can induce GDF15 expression.

In the liver, chemical induction of ER stress markedly increases *Gdf15* transcription and circulating GDF15 levels, a response driven by CHOP, which directly binds the *Gdf15* promoter and activates its transcription (67). In pancreatic β cells and human islets, ER stress also induces GDF15, but the regulation appears to involve C/EBPβ, suggesting that the upstream transcriptional program is cell type specific (68). In other tissues, such as skeletal muscle and liver during exercise or metabolic perturbation, GDF15 levels may rise even when ER stress signaling is not the dominant driver, indicating that ER stress is an important but not exclusive regulator of *Gdf15* (69). Thus, ER stress activates *Gdf15* in a context-dependent manner, with CHOP and C/EBPβ representing key transcriptional nodes.

Beyond its regulation by ER stress, GDF15 also modulates ER stress outcomes in a context-dependent manner: *Gdf15* deletion reduces ER stress-induced β-cell apoptosis and delays diabetes, whereas in other models GDF15 alleviates ER stress linked ferroptosis (67, 70). Overall, GDF15 is not merely a marker of ER stress; it can also actively modulate the biological outcome of ER stress signaling.

### 3.3 Oxidative stress

Oxidative stress is a fundamental feature of obesity and cardiometabolic disease, arising from nutrient excess, mitochondrial dysfunction, and chronic inflammation. GDF15 is strongly induced by oxidative stress, with the p53 pathway serving as a principal transcriptional regulator (71, 72). *In vivo* oxidative stress profiling has shown that *Gdf15* belongs to a conserved transcriptional response program activated under pro-oxidant conditions, together with canonical p53 target genes such as *p21*, *Gadd45a*, and *Trp53inp1* (73). In pulmonary epithelial and endothelial cells exposed to hyperoxia, GDF15 expression is markedly increased, whereas p53 knockdown significantly attenuates this induction (74). These findings indicate that GDF15 is not merely a downstream marker of cellular injury but rather part of an integrated p53 dependent stress response network (75).

Beyond its regulation by oxidative stress, GDF15 also orchestrates antioxidant defenses by forming a positive feedback loop with the master antioxidant transcription factor nuclear factor erythroid 2-related factor 2 (NRF2). Specifically, GDF15 activates the PI3K/AKT/GSK3β pathway to stabilize NRF2 protein, and NRF2 directly binds the *Gdf15* promoter to enhance its transcription, creating a self-amplifying circuit that maintains cellular redox balance (76). Functionally, GDF15 exerts direct antioxidant effects across multiple tissues. In models of myocardial infarction, GDF15 preserves mitochondrial function and suppresses reactive oxygen species (ROS) generation via AMP-activated protein kinase (AMPK) activation (77). As cardiomyocytes do not express GFRAL, these effects are mediated through non-GFRAL pathways, with AMPK activation representing a candidate downstream mechanism. In heart failure with preserved ejection fraction (HFpEF), *Gdf15* silencing leads to significant increases in oxidative stress, along with elevated inflammatory cytokines and apoptosis, indicating that endogenous GDF15 is required to contain oxidative injury (78).

However, the relationship between chronic GDF15 elevation and disease outcome is more complex. In chronic cardiometabolic disease states characterized by persistent oxidative stress, including obesity, type 2 diabetes, and heart failure, GDF15 levels become chronically elevated. This sustained elevation may initially represent a compensatory attempt to contain oxidative damage and maintain metabolic integrity. Yet, when the stress becomes unremitting, the same p53-GDF15 axis can contribute to maladaptive outcomes, including senescence-associated phenotypes such as p16-dependent endothelial senescence and ROS generation (79). Conversely, sustained *Gdf15* overexpression in transgenic mice has been shown to extend lifespan, accompanied by reduced IGF-1/insulin signaling and improved insulin sensitivity (80), indicating that chronic elevation is not uniformly detrimental. Thus, the pathological implications of chronic GDF15 elevation are context-dependent, shaped by the underlying disease state, the magnitude and duration of elevation, and the presence of concurrent pathological drivers. GDF15 functions as a context-dependent sentinel: its acute, reversible induction signals adaptive resilience to oxidative stress, whereas chronic elevation may mark either protective metabolic adaptation or pathological breakdown, depending on the broader physiological milieu.

### 3.4 Inflammatory stress

Inflammation is another common upstream trigger of GDF15 expression in multiple tissues and disease models (81, 82). Nuclear factor kappa B (NF-κB) is one of the major inflammatory transcription factors implicated in the regulation of GDF15 (83). This pattern suggests that GDF15 can function as a downstream response gene in NF-κB associated inflammatory networks. This is particularly important because obesity and MASLD are now recognized as inflammatory metabolic diseases, not merely disorders of excess fat mass (84).

The inflammatory induction of GDF15 may serve several purposes. In diabetic nephropathy, GDF15 overexpression suppressed NF-κB nuclear translocation through the IKK/IκBα pathway by interacting with NEDD4L (85). In LPS (lipopolysaccharide)-induced liver injury, GDF15 protected against inflammation by inhibiting TAK1 phosphorylation and subsequent NF-κB activation (86). In astrocytes, LPS-induced inflammatory stimulation altered IκB-α degradation and RelA/p65 phosphorylation, linking inflammatory activation to NF-κB-related regulation of GDF15 (81). This NF-κB-mediated feedback may limit tissue damage and modulate immune-metabolic communication, but chronic inflammation can lock GDF15 into a persistently elevated state reflecting disease burden rather than adaptive benefit.

### 3.5 Hypoxic stress

Hypoxia is increasingly recognized as an additional upstream factor in GDF15 regulation. Hypoxia is a fundamental feature of the tumor microenvironment and many ischemic or inflammatory tissues, and it activates the hypoxia-inducible factor 1-alpha (HIF1A) pathway to reprogram cellular transcriptional and metabolic responses (87). Beyond direct transcriptional regulation, hypoxia can also enhance GDF15 expression through intermediate stress pathways. In both renal cell and colon carcinoma cell lines, the hypoxia-inducible gene *Jmjd1a* regulates GDF15 expression under hypoxia by decreasing promoter histone methylation (88). In hepatocellular carcinoma, hypoxia-induced HIF1A upregulates the RNA m5C methyltransferase NSUN2, which stabilizes *Gdf15* mRNA via m5C modification and increases GDF15 secretion, revealing an additional post-transcriptional mechanism by which HIF1A controls GDF15 abundance (89). In colorectal cancer, chemotherapy-induced ROS activate HIF1A signaling in tumor cells and promote a tumor microenvironment that increases GDF15 production by tumor-associated macrophages, showing that HIF1A can also regulate GDF15 indirectly through immune cell crosstalk (90).

Functionally, the HIF1A–GDF15 axis is not purely linear, as GDF15 can feed back on HIF1A related pathways and modify downstream metabolic and inflammatory outputs. In sepsis-induced acute lung injury, GDF15 attenuates endothelial inflammation by suppressing the HIF-1α/LDHA pathway, thereby reducing glycolytic reprogramming and cytokine production (91). In sepsis associated liver injury, GDF15 promotes macrophage polarization from the M1 to the M2 phenotype by inhibiting the AMPK/PKM2/HIF1A axis, suggesting that GDF15 can counter-regulate HIF1A mediated inflammatory metabolism (92). In summary, hypoxia regulates GDF15 expression through multiple mechanisms, including decreased promoter histone methylation, RNA stabilization, and immune cell communication, while GDF15 in turn modulates HIF1A associated metabolic and inflammatory pathways to shape disease outcomes.

In summary, GDF15 is not regulated by a single stress pathway but by the convergence of ISR, ER stress, oxidative stress, inflammation, and hypoxia onto shared transcriptional nodes including ATF4, CHOP, p53, NRF2, NF-κB, and HIF1A. Acute, reversible stress drives transient GDF15 elevation supporting adaptation, whereas chronic stress leads to sustained elevation marking organ dysfunction. Interpreting GDF15 thus requires considering stress duration and magnitude, not merely absolute levels (**Figure 2**).

**Figure 2:**
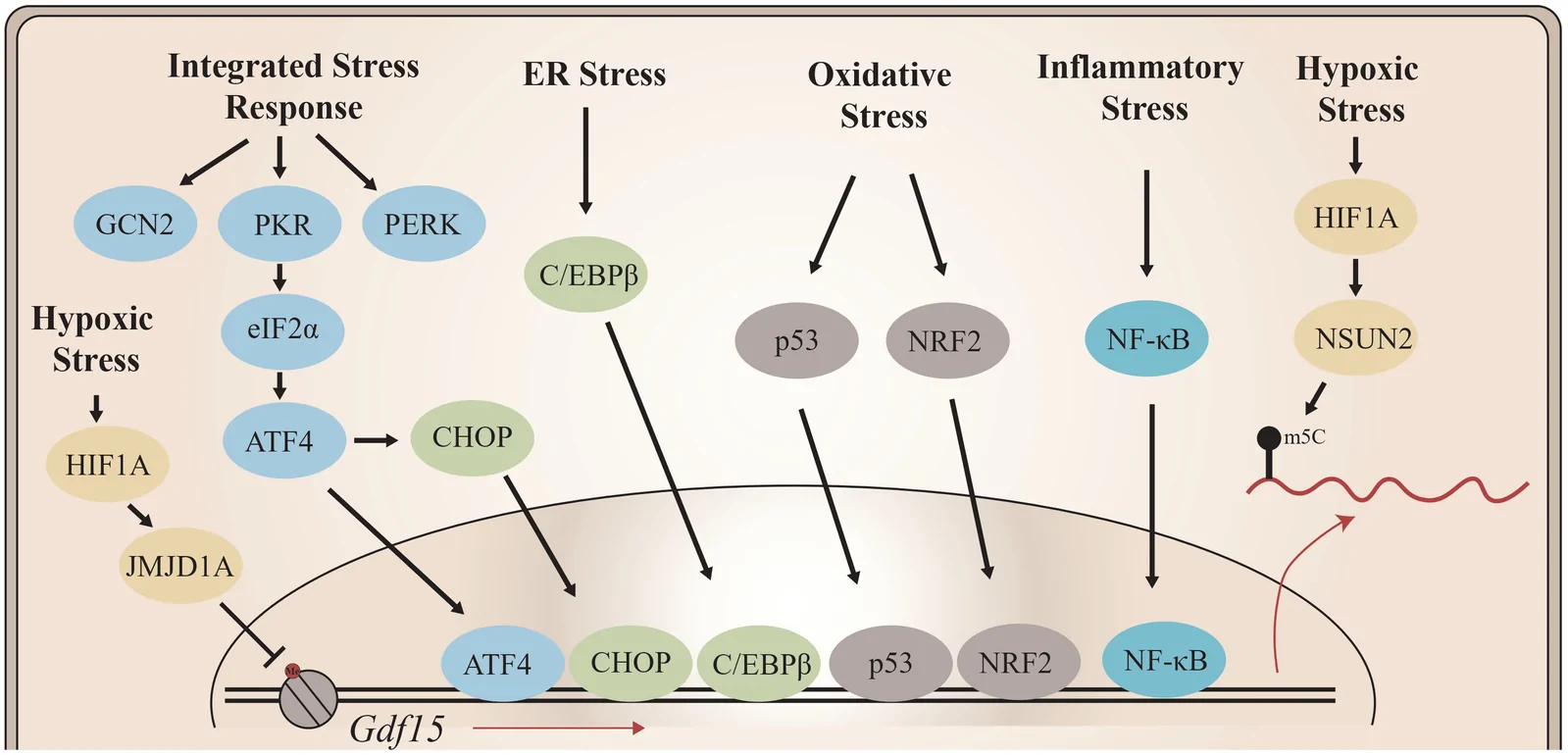
Convergent stress signaling pathways regulating GDF15 expression. Multiple convergent stress signaling pathways, including ISR (integrated stress response), ER stress (endoplasmic reticulum stress), oxidative stress, inflammatory stress, and hypoxic stress, transcriptionally and post-transcriptionally regulate *Gdf15* expression. ISR is activated via GCN2 (general control nonderepressible 2), PKR (protein kinase R), and PERK (protein kinase RNA-like endoplasmic reticulum kinase), leading to eIF2α (eukaryotic translation initiation factor 2α) phosphorylation and subsequent ATF4 (activating transcription factor 4) and CHOP (C/EBP homologous protein) activation. ER stress also engages the PERK-eIF2α-ATF4-CHOP axis, with additional involvement of C/EBPβ. Oxidative stress induces GDF15 through p53 and NRF2 (nuclear factor erythroid 2-related factor 2) dependent mechanisms. Inflammatory stress signals through NF-κB (nuclear factor kappa B) to upregulate GDF15 transcription. Hypoxic stress activates HIF1A (hypoxia-inducible factor 1-alpha), which can indirectly enhance GDF15 expression via JMJD1A (Jumonji domain-containing protein 1A) mediated histone demethylation or NSUN2 (NOP2/Sun RNA methyltransferase 2) mediated m^5^C RNA stabilization. These multiple stress-responsive transcriptional and post-transcriptional regulators converge on the *Gdf15* gene, positioning GDF15 as a systemic sentinel that integrates diverse cellular insults into a coordinated endocrine output.

## 4 Multi-organ sources of GDF15

GDF15 is a stress-responsive cytokine whose expression is induced across multiple tissues in response to diverse insults, including mitochondrial dysfunction, oxidative stress, hypoxia, inflammation, metabolic imbalance, and genotoxic injury. This multi-organ expression pattern positions GDF15 as a key integrator of local stress signals into systemic metabolic, inflammatory, and behavioral responses.

The evidence for multi-organ GDF15 expression derives from diverse experimental approaches that offer different levels of inference. Tissue expression profiling (mRNA and protein detection in human or rodent tissues) establishes the presence of GDF15 but does not indicate its functional significance. Genetic loss- or gain-of-function studies in mice provide causal evidence for the contribution of a given tissue to circulating GDF15 pools. Pharmacological intervention studies (recombinant protein or neutralizing antibodies) inform therapeutic potential. Throughout this section, we have indicated the nature of the evidence for each tissue source to facilitate interpretation of the relative contributions of different organs across physiological and pathological states.

### 4.1 Adipose tissue

In obesity, adipose tissue is a key source of circulating GDF15 at disease onset, with expression predominantly induced in white adipose tissue (WAT) in association with macrophage accumulation; macrophages are the main immune cells expressing GDF15 in this setting (tissue expression profiling) (93). Macrophage specific inactivation of *Gdf15* reduces plasma GDF15 levels and paradoxically exacerbates obesity (genetic evidence), indicating that macrophage derived GDF15 serves a compensatory protective function against diet-induced weight gain (93).

Beyond its role in obesity, GDF15 is also required for maintaining subcutaneous adipose tissue (sWAT) lipid metabolic homeostasis. *Gdf15* KO mice exhibit broad downregulation of *de novo* lipogenesis enzymes in sWAT, with female mice being more severely affected, and fasting induces GDF15 expression in sWAT of both mice and humans (94). Additionally, adipocyte derived GDF15 preferentially suppresses high fat diet (HFD) intake via GFRAL signaling, improving obesity and glucose tolerance (66).

Under psychological stress, β-adrenergic signaling in WAT induces lipolysis, and the resulting free fatty acids (FFAs) stimulate M2-like macrophages within WAT to secrete GDF15; this GDF15 then acts through GFRAL to elicit anxiety-like behavior, providing a direct molecular link between adipose tissue metabolism and behavior (95). Furthermore, in a mouse model of ovarian cancer cachexia, serum GDF15 levels increase significantly during cachexia progression, accompanied by dramatic fat loss and upregulation of thermogenic genes such as uncoupling protein 1 (*Ucp1*) in adipose tissue (96). Thus, adipose tissue functions as a dynamic stress sensor, with macrophages serving as the primary GDF15 producing cells under metabolic and psychological stress, and this adipose tissue derived GDF15 acts both locally to maintain lipid homeostasis and systemically to regulate appetite and behavior.

### 4.2 Skeletal muscle

Skeletal muscle is a critical source of stress-inducible GDF15, functioning as a myokine that mediates both local and systemic metabolic adaptations. GDF15 expression in skeletal muscle can be robustly induced by various stressors, including mitochondrial dysfunction, oxidative stress, ER stress, exercise, and aging (97). In middle aged mice, *Gdf15* expression is selectively increased in skeletal muscle, but not in liver, adipose tissue, kidney, or heart, and this elevation correlates with impaired mitochondrial respiratory capacity and insulin sensitivity (tissue expression profiling and correlative analysis). These findings identify skeletal muscle as a major source of age-related circulating GDF15 elevation, although formal causal demonstration awaits muscle-specific genetic manipulation studies (98).

Mechanistically, mitochondrial dysfunction in skeletal muscle activates the ISR, leading to GDF15 induction. In mice with muscle-specific mitochondrial uncoupling (*Ucp1*-TG), GDF15 is induced as a myokine with diurnal variation, and genetic loss of *Gdf15* abolishes systemic metabolic flexibility, insulin sensitivity, and WAT browning, while promoting progressive fat mass gain (38). Lactate driven histone lactylation has been identified as an epigenetic mechanism linking mitochondrial stress to GDF15 secretion in skeletal muscle under mtDNA mutation-induced mitochondrial myopathy (99). In patients with primary mitochondrial myopathy, elevated circulating GDF15 is associated with muscle atrophy and exercise intolerance (observational association in humans). Importantly, in a mouse model of mitochondrial myopathy, GDF15 neutralization with a monoclonal antibody improves body weight, lean mass, muscle force production, and voluntary wheel running, reversing transcriptional dysregulation in autophagy and proteasome signaling pathways (pharmacological intervention in animals), suggesting therapeutic potential that warrants clinical validation (100).

The biological outcome of muscle derived GDF15 is context-dependent, following a hormetic pattern: under acute stressors such as exercise, GDF15 exhibits protective effects, promoting metabolic homeostasis and muscle health; conversely, when chronically expressed in pathological processes such as aging, obesity, or chronic inflammation, GDF15 correlates with the degree of muscle impairment and mitochondrial stress (97). In cancer cachexia, tumor derived factors induce GDF15 expression, and neutralization of GDF15 restores muscle mass and physical performance in preclinical models (101). This translational potential has been recently validated in a phase 2 randomized controlled trial, in which ponsegromab, a humanized monoclonal antibody that neutralizes GDF15, significantly increased body weight, improved appetite and cachexia symptoms, and enhanced physical activity in patients with cancer cachexia and elevated circulating GDF15 levels, establishing GDF15 as a therapeutically targetable driver of this debilitating condition (102). In humans, circulating GDF15 levels increase with age and are inversely associated with muscle mass and strength, particularly in older adults (103, 104).

### 4.3 Liver

The liver is a primary source of stress-inducible GDF15, particularly in response to metabolic dysfunction, chemical toxicity, inflammation, mitochondrial dysfunction, and malignant transformation. In models of chemical-induced liver injury, such as carbon tetrachloride (CCl_4_) or alcohol exposure, GDF15 expression is robustly induced in the liver. Genetic ablation of *Gdf15* exacerbates inflammation and fibrosis (loss-of-function evidence), whereas hepatocyte-specific *Gdf15* overexpression or recombinant GDF15 administration alleviates injury (gain-of-function and pharmacological evidence), collectively indicating a non-redundant protective function (105, 106). Furthermore, the widely prescribed anti-diabetic drug metformin directly induces hepatic GDF15 secretion through AMPK- and PPARβ/δ-dependent pathways, and this liver derived GDF15 is essential for mediating the drug’s appetite suppressing and weight loss effects via GFRAL (32, 107, 108).

Beyond metabolic and toxic stress, the liver serves as a critical source of GDF15 during inflammatory, infectious, and hypoxic insults. In sepsis and endotoxemia, the liver rapidly produces GDF15, which reprograms macrophage metabolism via the AMPK/PKM2/HIF1A pathway to dampen hyperinflammatory responses and promote disease tolerance (109, 110). In chronic hepatitis C and B virus infections, hepatocyte GDF15 expression is significantly elevated, and circulating levels correlate with the degree of liver fibrosis, disease severity, and the subsequent risk of developing hepatocellular carcinoma (HCC) after viral clearance (111–113). Under conditions of mitochondrial dysfunction, such as primary mitochondrial hepatopathies or the mitochondrial UPR, the liver secretes GDF15 as a bona fide mitokine, which, in conjunction with fibroblast growth factor 21 (FGF21), serves as a sensitive diagnostic biomarker for mitochondrial liver diseases (39, 114, 115).

In the context of malignant transformation and ischemia-reperfusion injury, liver derived GDF15 adopts a predominantly pathogenic role. In HCC, GDF15 is highly overexpressed by cancer cells (tissue expression profiling in human tumors), and *in vitro* and xenograft studies have demonstrated that GDF15 promotes tumor growth, invasion, and metastasis through activation of the AKT/GSK-3β/β-catenin pathway, while also fostering an immunosuppressive tumor microenvironment (TME) by expanding regulatory T cells (Tregs) and suppressing dendritic cell maturation (DC) (cell culture and animal tumor models) (46, 116, 117). This immunosuppressive effect is mediated in part through GDF15-CD48 signaling, which promotes regulatory T cell generation and suppressive function. Clinically, serum GDF15 is a potent diagnostic and prognostic biomarker for HCC, and its combination with alpha-fetoprotein (AFP) significantly improves the accuracy of early stage HCC detection (111, 118). In hepatic ischemia–reperfusion injury, GDF15 is among the most highly upregulated genes, suggesting its involvement in the pathophysiological response to hypoxic stress (119). Thus, the liver functions as a central stress sensor, and hepatic GDF15 serves as a critical integrator of local metabolic, toxic, inflammatory, and malignant signals, acting as a protective endogenous mediator, a driver of pathology, and a useful clinical biomarker depending on context.

### 4.4 Heart

The heart is a prominent source of GDF15 under ischemic and hemodynamic stress. In myocardial infarction (MI), GDF15 expression is significantly upregulated in the infarcted myocardium, and *Gdf15* deficiency aggravates cardiac injury (77). Cardiac GDF15 preserves mitochondrial function and suppresses hypoxia-induced ROS generation through AMPK activation; exogenous GDF15 administration reduces myocardial injury and fibrosis in rodent models (pharmacological intervention) (77), though whether these effects are mediated via central GFRAL signaling or through yet-unidentified peripheral receptors on cardiomyocytes remains unclear. During exercise training, physiological cardiac adaptation requires suppression of cardiomyocyte GDF15 via peroxisome proliferator activated receptor gamma coactivator 1 alpha (PGC-1α) (120).

Cardiomyocytes and cardiac fibroblasts are also the primary sources of GDF15, with their expression strongly induced by various pathological stimuli. In rodent models of MI, ischemia/reperfusion (I/R) injury, and pressure overload, *Gdf15* mRNA and protein levels in cardiomyocytes increase rapidly within hours, involving transcription factors such as ATF3 (*in vivo* animal models and *in vitro* evidence) (121). Neurohormones such as angiotensin II and norepinephrine can also directly stimulate GDF15 production in isolated cardiomyocytes (ex vivo/*in vitro*) (122). Cardiac fibroblasts are another significant source of GDF15, particularly in models of radiation-induced heart disease or pressure overload, where ionizing radiation upregulates GDF15 expression via the MAPK/ERK1/2 pathway, subsequently promoting cardiac fibrosis (cell culture and animal models) (123). Furthermore, GDF15 blocks norepinephrine induced cardiac hypertrophy by inhibiting EGFR transactivation and antagonizes pressure overload induced hypertrophy via the SMAD2/3 pathway (122, 124). Despite these protective findings in basic science (animal models and *in vitro* studies), numerous clinical studies demonstrate that persistently high circulating GDF15 levels are strongly associated with adverse outcomes, left ventricular (LV) remodeling, and myocardial fibrosis in patients with HF (observational association) (125, 126). This apparent paradox, protective in acute animal models but prognostic of poor outcomes in chronic human disease, underscores the context-dependent nature of GDF15 signaling and highlights that the functional implications of GDF15 elevation depend on disease stage, chronicity, and the underlying metabolic and age-related milieu.

### 4.5 Kidney

The kidney is a critical source and regulator of stress inducible GDF15, particularly in response to metabolic, pharmacological, and genotoxic stress. Notably, the kidney produces GDF15 but does not express its cognate receptor GFRAL, indicating that renal GDF15 functions as an endocrine signal rather than a local autocrine/paracrine factor. In obesity and type 2 diabetes (T2D), the kidney is a major contributor to elevated circulating GDF15. FFAs directly increase renal *Gdf15* mRNA expression in rodent models, with expression localized to the renal cortex and outer medulla (*in vivo* and ex vivo evidence). Furthermore, kidney-derived GDF15 is necessary for FFA-induced suppression of food intake, as demonstrated by kidney-specific Gdf15 knockdown studies (genetic evidence) (127, 128), although the precise quantitative contribution of renal versus extra-renal GDF15 sources remains to be determined.

Metformin increases plasma GDF15 levels primarily through upregulation of GDF15 synthesis in the kidney; kidney specific knockdown of *Gdf15* negates metformin’s ability to lower food intake and body weight (129). This renal GDF15 production is mediated by PPARβ/δ and AMPK (107). Additionally, in a mouse model of Cockayne syndrome, endogenous formaldehyde driven transcriptional stress induces GDF15 expression specifically in a subset of kidney proximal tubule cells, and this renal GDF15 drives cachexia and anorexia; blocking this response with an anti-GDF15 antibody alleviates the wasting syndrome (130, 131). Of note, single-cell or spatial transcriptomic evidence localizing GDF15 production to specific human nephron segments is currently lacking, and most available data derive from rodent models or bulk tissue analyses.

Beyond its role as an endocrine signal, kidney derived GDF15 serves as a potent early biomarker of renal injury and a critical mediator of nephroprotection. In acute kidney injury (AKI) models, such as unilateral IRI or cisplatin-induced nephrotoxicity, serum and urinary GDF15 levels are rapidly elevated within hours, primarily originating from injured proximal tubular cells (biomarker evidence in animal models) (132, 133). Functionally, mouse genetic studies demonstrate that GDF15 plays a renoprotective role: *Gdf15*-deficient mice exhibit exacerbated tubular injury, enhanced inflammation, and more severe fibrosis in models of AKI, diabetic nephropathy, and UUO (genetic loss-of-function evidence), whereas GDF15 overexpression or recombinant GDF15 administration preserves kidney function, reduces apoptosis, and limits fibrosis (genetic gain-of-function and pharmacological evidence) (134–137). In humans, elevated circulating GDF15 correlates with renal injury and predicts adverse kidney outcomes, though these associations do not establish causality. Mechanistically, GDF15 preserves the expression of the nephroprotective factor Klotho in tubular cells, promotes autophagy and lysosome biogenesis via inhibition of the PI3K/Akt/mTOR pathway, and induces an anti-inflammatory M2 macrophage phenotype to limit transplant injury (107, 138, 139).

### 4.6 Immune cells

Macrophages are a major source of stress inducible GDF15 and also a key target of its immunomodulatory actions. The immunomodulatory function of GDF15 was first identified by Bootcov et al., who demonstrated that recombinant GDF15 directly suppresses TNFα production from LPS stimulated macrophages, establishing GDF15 (then named MIC-1) as a negative regulator of inflammatory responses (20). Under inflammatory conditions, such as exposure to LPS or IFN-γ, macrophages upregulate GDF15 expression *in vitro*, and this GDF15 in turn feeds back to limit excessive inflammation by suppressing TNFα production (cell culture evidence) (20). During oxidative and lysosomal stress, macrophages produce high levels of GDF15, which has been implicated in promoting fibrogenesis and angiogenesis *in vitro* and in animal models, while also switching the macrophage phenotype toward an M2-like state (*in vitro* and *in vivo* evidence) (140). GDF15 also potently modulates macrophage polarization: it directly induces human CD14^+^ monocytes to adopt an M2-like phenotype *in vitro*, inhibits M1-like polarization, and promotes anti-inflammatory properties through metabolic reprogramming toward oxidative phosphorylation (OXPHOS) (cell culture evidence) (105, 141). In animal models of liver fibrosis and sepsis-associated liver injury, GDF15 ameliorates disease by promoting M2 macrophage polarization and suppressing pro-inflammatory cytokine release (*in vivo* pharmacological and genetic evidence) (92, 105). Similarly, in a mouse model of diabetic cardiomyopathy, GDF15 treatment alleviated diastolic dysfunction and reduced cardiac inflammation and macrophage infiltration independent of weight loss (142), further supporting GDF15’s anti-inflammatory actions *in vivo*. Conversely, in certain TMEs, such as ovarian cancer, GDF15 facilitates immune escape by suppressing DC function, while in oral squamous cell carcinoma (OSCC), macrophage intrinsic GDF15 enhances phagocytosis of tumor cells and cross-presentation to CD8^+^ T cells, exerting anti-tumor effects (143, 144). These findings highlight the context-dependent and cell-autonomous roles of GDF15 in macrophage biology.

### 4.7 Intestine

Under basal conditions, GDF15 expression in the intestinal mucosa is low but detectable, with prominent synthesis occurring in epithelial cells of the small and large intestine (145). In mice, oral metformin increases GDF15 expression in the distal intestine, and deletion of *Gdf15* from the intestinal epithelium significantly reduces the circulating GDF15 response to oral metformin (146). Mechanistically, metformin inhibits mitochondrial complex I in intestinal epithelial cells, leading to mitochondrial dysfunction and subsequent GDF15 upregulation (147, 148).

Intestinal GDF15 expression is potently induced during microbial infections and inflammatory conditions. In patients with acute cholera, genome-wide microarray analysis of duodenal mucosa revealed marked upregulation of GDF15 as part of a broad innate defense response (human tissue transcriptomics) (149). Infection of intestinal epithelial cells with enteropathogenic E. coli (EPEC) induces GDF15 expression through the stress-regulated transcription factor CHOP (*in vitro* evidence) (150). Infection with the opportunistic fungus *Candida albicans* also triggers GDF15 expression in intestinal epithelial cells via NF-κB signaling *in vitro*, and inhibition of NF-κB activation exacerbates epithelial cytotoxicity, indicating a protective role for GDF15 in the host response to fungal challenge (cell culture evidence) (151). In inflammatory bowel disease (IBD), circulating GDF15 levels are elevated in patients with Crohn’s disease and ulcerative colitis, correlating with markers of inflammation and intestinal permeability (152). Mechanistically, GDF15 directly alters the intestinal barrier by decreasing the expression of tight junction proteins, thereby increasing paracellular permeability (152).

The intestinal epithelium also produces GDF15 in response to metabolic and nutritional challenges. In mice fed an HFD, intestinal epithelial cell-specific deletion of *Raptor* (a core component of mTORC1) leads to reduced food intake and protection against diet-induced obesity, accompanied by markedly enhanced GDF15 expression in enterocytes (153). Protein energy malnutrition (PEM) induces long-lasting epigenetic changes in the intestinal epithelium, including persistent upregulation of GDF15 expression driven by PPAR signaling. This sustained GDF15 elevation is linked to depletion of Paneth cells and suppression of antimicrobial gene expression, changes that persist despite dietary recovery and are dependent on the gut microbiota, particularly microbial lipid metabolites such as 9-HODE (154).

Beyond its role as a stress-induced systemic hormone, GDF15 exerts important local effects on intestinal epithelial cell fate. GDF15 is highly expressed in the colonic surface epithelium, where cells undergo apoptosis, and its expression is reduced in colorectal tumors compared to normal tissue (155). During ulcerative mucosal injury, GDF15 promotes early epithelial restitution by enhancing enterocyte migration and spreading (156). Genetic ablation of *Gdf15* decreases survival in a mouse model of intestinal ulcer and delays epithelial restitution, confirming its protective role in wound healing (156). However, under certain conditions, such as exposure to the food additive carrageenan, GDF15 can also promote epithelial cell apoptosis, with the pro-survival protein ATF3 counteracting this effect (157).

### 4.8 Lung

Under acute inflammatory stress, such as sepsis or acute lung injury (ALI), the lung epithelium is a major source of GDF15. In critically ill patients, lower respiratory tract GDF15 levels correlate with plasma levels and are higher in non-survivors (observational association in humans). In mouse models, intratracheal administration of bacterial exoproducts induces airspace and plasma release of GDF15, an effect that is attenuated with epithelial specific deletion of *Gdf15* (genetic evidence in animals), confirming the lung epithelium as a source of stress inducible GDF15 (158). Similarly, in models of hyperoxic lung injury, *Gdf15* deficiency exacerbates mortality and disrupts alveolarization, indicating a non-redundant protective function for GDF15 in the developing lung (159). In IPF, senescent alveolar type II epithelial cells are the primary source of GDF15, with markedly increased expression in lung tissue and elevated circulating levels that correlate with disease severity and poor survival (human tissue profiling and observational association) (160, 161). Mechanistic studies in cell culture have shown that epithelial derived GDF15 promotes fibrosis by activating lung fibroblasts and inducing myofibroblast differentiation via the ALK5-Smad2/3 pathway (*in vitro* evidence), and its neutralization in animal models significantly reduces bleomycin-induced lung fibrosis (pharmacological intervention in animals) (162, 163). In COPD, cigarette smoke exposure induces GDF15 production in airway epithelial cells, which in turn promotes cellular senescence and pulmonary inflammation, contributing to disease pathogenesis and acute exacerbations (164).

In lung cancer, GDF15 is highly expressed by tumor cells and cancer associated fibroblasts (CAFs) within the TME, where it drives malignancy and immune evasion. Tumor derived GDF15 promotes non-small cell lung cancer (NSCLC) proliferation, invasion, and metastasis through the PI3K/AKT and TGF-β/Smad pathways, and contributes to resistance to both chemotherapy and immunotherapy (165–167). Single cell analyses reveal that GDF15 is a key hub in the immunosuppressive microenvironment, and elevated GDF15 expression in tumors correlates with poor response to PD-1/PD-L1 inhibitors (168, 169). In patients with NSCLC with *STK11*/*LKB1* mutations, tumor derived GDF15 is a critical mediator of cancer cachexia, and GDF15 neutralizing antibodies are currently in clinical trials for the treatment of cancer cachexia and checkpoint inhibitor resistance (102, 167).

### 4.9 Central nervous system

Within the central nervous system (CNS), GDF15 is expressed by multiple cell types, including neurons, astrocytes, microglia, and the choroid plexus epithelium, with expression dramatically induced following injury or stress. In models of cerebral ischemia, *Gdf15* mRNA and protein are strongly upregulated in hippocampal and cortical neurons as early as 3 hours after middle cerebral artery occlusion (170). Similarly, following optic nerve crush, GDF15 expression increases in the murine retina (171). The choroid plexus is the major constitutive source of GDF15 in the brain, secreting the protein into the cerebrospinal fluid (CSF), from which it can penetrate the parenchyma (145). In Alexander disease, a severe neurodegenerative disorder caused by GFAP mutations, astrocytes are the primary source of elevated GDF15 in the brainstem and CSF, where it correlates with failure to thrive and anorexia (172). Under conditions of mitochondrial dysfunction, such as in vanishing white matter disease, the ISR within CNS cells drives GDF15 expression, making CSF GDF15 a dynamic pharmacodynamic biomarker for ISR-activating therapies (173).

GDF15 exerts pleiotropic effects on neuroinflammation and neuronal survival. In the 6-hydroxydopamine mouse model of Parkinson’s disease, GDF15 deficiency exacerbates dopaminergic neuron loss and blunts the microglial response, indicating a direct neurotrophic and immunomodulatory role (174). Conversely, GDF15 promotes survival of spinal and facial motoneurons, and *Gdf15* deficient mice exhibit progressive postnatal loss of these neurons (175).

Elevated GDF15 levels in CSF or serum are associated with a wide spectrum of CNS disorders. In AD, plasma GDF15 is associated with increased dementia risk and brain aging in large cohort studies, and GDF15 levels in neuronal-derived EVs can discriminate AD patients from controls (observational association in humans) (176, 177). In a mouse model of ALS, high serum GDF15 contributes to weight loss and altered lipid metabolism, and GFRAL silencing in the AP/NTS prolongs survival (genetic evidence in animals) (178). In primary mitochondrial disorders, CSF GDF15 is a sensitive marker of CNS involvement and the ISR (179, 180). GDF15 is also elevated in multiple system atrophy, glioma, and epilepsy, where it correlates with disease severity and progression (181–183).

### 4.10 Vascular endothelium

The vascular endothelium is both a significant source and a critical target of stress inducible GDF15, particularly in response to inflammatory, metabolic, mechanical, and cytotoxic stimuli. Under pro-inflammatory conditions, such as those mimicked by a cytokine mixture, human umbilical vein endothelial cells upregulate GDF15 *in vitro*, which then acts in an autocrine manner to promote endothelial-to-mesenchymal transition, thereby reducing the anticoagulant and fibrinolytic capacity of the endothelium (cell culture evidence) (184). In patients with anti-PR3-positive vasculitis, neutrophil derived extracellular vesicles induce GDF15 secretion from human umbilical vein endothelial cells (HUVECs), alongside other inflammatory mediators like Interleukin-8 and vascular cell adhesion molecule-1 (VCAM-1), recapitulating the pro-inflammatory endothelial phenotype seen in autoimmune vasculitis (185). Furthermore, thoracic X-irradiation (a model for radiotherapy-induced cardiovascular damage) induces GDF15 secretion from coronary artery and microvascular endothelial cells *in vitro* and elevates serum GDF15 levels in *ApoE*^-/-^ mice, identifying GDF15 as a potential early biomarker of radiation-induced vascular injury (186).

In addition to being a source, the vascular endothelium is a primary target of GDF15’s modulatory effects on vascular tone and function. Exogenous GDF15 directly impairs aortic contractile and endothelium-dependent relaxing functions in ex vivo vascular ring preparations by disrupting caveolar endothelial nitric oxide synthase (eNOS) signaling, leading to its dissociation from caveolin-1 and reducing nitric oxide (NO) bioavailability (ex vivo evidence) (187). The endothelial receptor for GDF15 remains unknown. In patients with type 2 diabetes, elevated GDF15 levels are independently associated with increased arterial stiffness and impaired endothelium dependent vasodilation (observational association in humans), consistent with the vascular effects observed in ex vivo studies (188). Paradoxically, while GDF15 can induce dysfunction under pathological conditions, exogenous GDF15 administration has been shown to protect against diabetic endothelial dysfunction by activating AMPK, reducing oxidative stress, suggesting a context-dependent, potentially protective role (189). In the setting of aging, perivascular adipose tissue (PVAT) from aged mice impairs endothelial function in young aortas partially through GDF15, while young PVAT can rejuvenate aged aortas, highlighting the role of PVAT-derived GDF15 in vascular aging (190).

Finally, the endothelium is involved in the polarized secretion of GDF15, which is released preferentially into the basolateral medium from human microvascular endothelial cells, indicating a directionally specific signaling role (191). Clinically, elevated circulating GDF15 is associated with markers of endothelial activation (such as vWF, ICAM-1, and VCAM-1) and vascular pathologies, including aortic valve stenosis and atherosclerosis (192–194). In ultramarathon runners, extreme exercise induces a transient rise in GDF15 along with NT-proBNP and endoglin, reflecting acute endothelial dysfunction (195).

Taken together, circulating GDF15 reflects a weighted sum of tissue specific contributions that vary by disease state: adipose tissue macrophages in early obesity, skeletal muscle in aging and mitochondrial disorders, kidney and intestine in metformin response, and the diseased organ itself in advanced liver or heart failure. However, it is important to note that the strength of evidence for each tissue source differs: some contributions have been established through tissue specific genetic manipulation (e.g., kidney, intestine, macrophages), whereas others are inferred primarily from tissue expression profiling and correlative studies (e.g., heart, lung, vasculature). No single source dominates across all conditions. This has two implications: circulating GDF15 is an integrated readout of systemic stress burden, not a specific organ marker; and therapeutic modulation of GDF15 must consider which tissue source is dominant in a given disease state to avoid unintended inter-organ effects (**Figure 3**).

**Figure 3:**
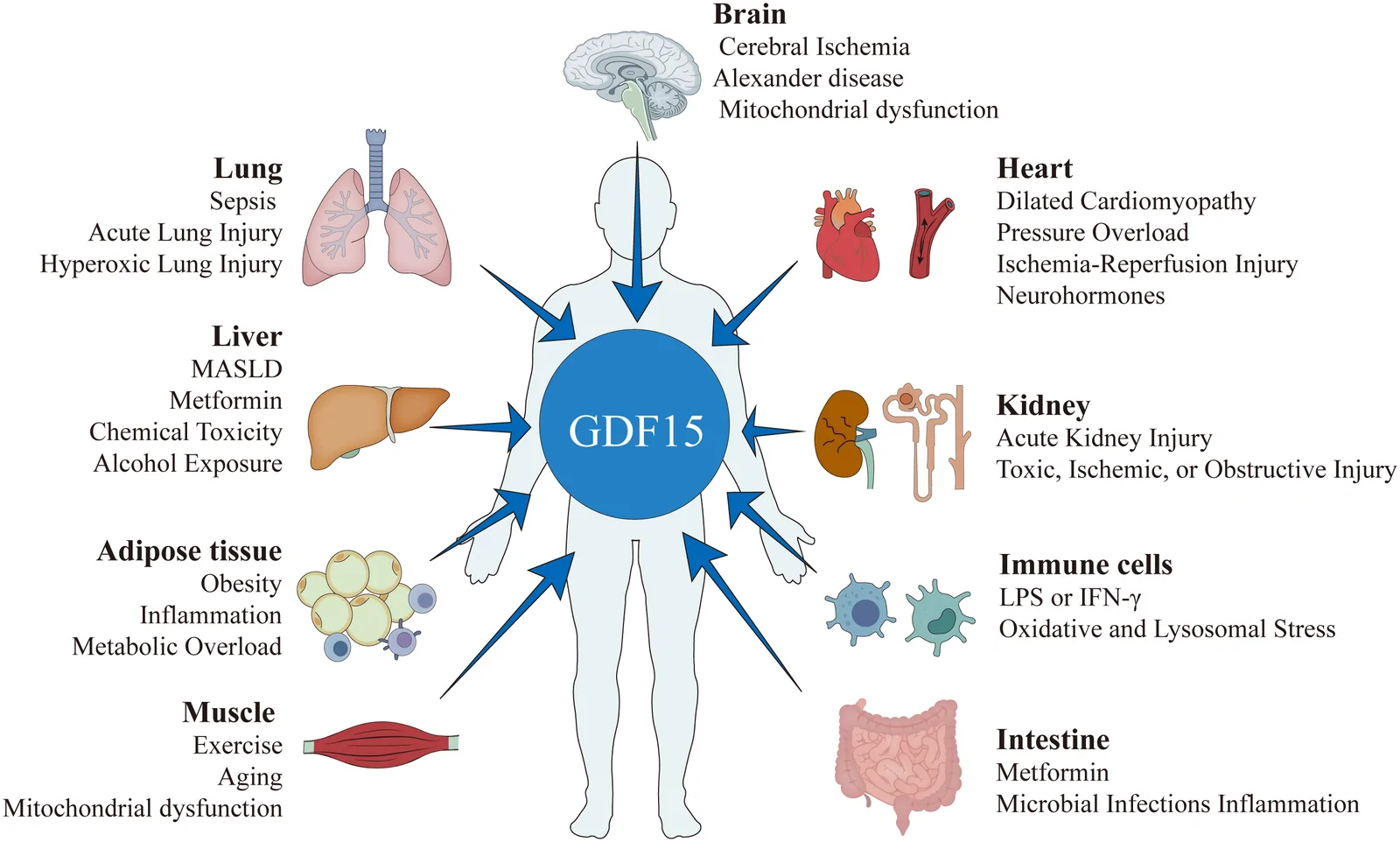
Multi-organ sources of stress-inducible GDF15 under diverse pathological and physiological conditions. GDF15 is induced by diverse stressors across multiple organs, highlighting its role as a key integrator of local stress signals into systemic responses. In the brain, GDF15 is induced by cerebral ischemia, Alexander disease, and mitochondrial dysfunction. In the lung, sepsis, ALI (acute lung injury), and hyperoxic lung injury trigger GDF15 expression. In the heart, dilated cardiomyopathy, pressure overload, I/R (ischemia-reperfusion) injury, and neurohormonal stimulation upregulate GDF15. In the liver, MASLD (metabolic dysfunction-associated steatotic liver disease), metformin exposure, chemical toxicity, and alcohol exposure induce hepatic GDF15. In the kidney, AKI (acute kidney injury), as well as toxic, ischemic, or obstructive injury, stimulate renal GDF15 production. In the adipose tissue, obesity, inflammation, and metabolic overload drive GDF15 expression, particularly in adipose tissue macrophages. In the immune cells: inflammatory stimuli such as LPS (lipopolysaccharide) or IFN-γ (interferon-gamma), along with oxidative and lysosomal stress, induce GDF15 in macrophages and other immune cells. In the Muscle: Exercise, aging, and mitochondrial dysfunction promote GDF15 as a myokine. In the intestine, metformin, microbial infections, and inflammation trigger GDF15 production from intestinal epithelium. This multi-organ expression pattern positions GDF15 as a key integrator of local stress signals into systemic metabolic, inflammatory, and behavioral responses.

## 5 GDF15 as an adaptive effector preserving metabolic integrity

Having established the multi-organ sources of GDF15 in Section 4, this section focuses on its pathophysiological roles in specific disease contexts. Throughout this section, we interpret the evidence through the lens of the sentinel hypothesis. However, it is important to emphasize that the distinction between adaptive and maladaptive GDF15 elevation is currently descriptive rather than mechanistically defined. In most cases, the same study that reports protective effects of GDF15 in an acute model also reports associations between chronic elevation and poor outcomes in human cohorts. Whether these represent opposite ends of a continuum or entirely distinct biological processes remains unclear. We therefore present the acute-adaptive versus chronic-maladaptive framework as an organizing principle for hypothesis generation, not as a proven biological dichotomy.

Before examining the role of GDF15 in specific disease contexts, it is important to recognize that the supporting evidence derives from distinct experimental paradigms that confer different levels of causal inference. Human observational studies establish associations between circulating GDF15 levels and disease prevalence or prognosis but cannot distinguish whether GDF15 is causal, compensatory, or merely a marker of disease burden. By contrast, mouse genetic loss- and gain-of-function studies provide direct causal evidence for GDF15’s biological actions, while pharmacological intervention studies (e.g., recombinant protein or neutralizing antibodies) inform therapeutic potential. Throughout the following sections, we have indicated the nature of the evidence for each claim, observational, genetic, or pharmacological, to clarify the strength and limitations of the current knowledge base.

### 5.1 Obesity

*Gdf15* expression is robustly upregulated in response to multiple metabolic stressors associated with obesity, including HFD feeding and saturated FFAs (67, 196). It is important to recognize that the biological outcome of GDF15 elevation is modified by host factors such as age and metabolic context. Older adults exhibit higher baseline GDF15 levels and may have blunted responses to its anorectic effects, and the same absolute GDF15 concentration may have different implications in metabolically healthy versus obese or diabetic individuals. These modifying factors are not captured by a simple acute-versus-chronic dichotomy.

In diet-induced obese (DIO) mice, circulating GDF15 levels increase progressively with sustained HFD feeding (observational association). Genetic loss of *Gdf15* exacerbates weight gain (causal evidence), indicating that endogenous GDF15 serves as an adaptive response to nutrient excess (63). Furthermore, hepatocytes also contribute to elevated GDF15 levels in obese states, as liver specific *Gdf15* expression is sufficient to drive circulating GDF15 changes (197). This induction positions GDF15 as an adaptive effector that senses metabolic overload and attempts to restore energy balance.

Once elevated, GDF15 acts through its hindbrain-restricted receptor GFRAL to reduce food intake and promote weight loss, thereby directly counteracting the positive energy balance that drives obesity (13, 198). Notably, beyond its appetite suppressing effects, GDF15 enhances energy expenditure by increasing fatty acid oxidation and calcium futile cycling in skeletal muscle, preventing the adaptive thermogenic reduction that often accompanies diet induced weight loss (40). Pharmacological GDF15 administration also improves insulin sensitivity in obese rodents through a β-adrenergic-dependent mechanism, enhancing glucose uptake in adipose tissue and suppressing hepatic glucose production (pharmacological intervention), suggesting that exogenous GDF15 can recapitulate and amplify its endogenous adaptive functions (199). In addition, GDF15 inhibits adipogenesis by enhancing HOP2 expression and suppressing C/EBPα, thereby limiting AT expansion (200). These multifaceted actions collectively preserve metabolic integrity in the face of sustained caloric excess.

Beyond its effects on food intake and energy expenditure, GDF15 has been proposed to influence body weight through additional mechanisms that may have important clinical implications. Preclinical studies have shown that GDF15 can alter food preference and induce conditioned taste aversion, suggesting that its anorectic effects are at least partially mediated through aversive visceral malaise rather than purely homeostatic satiety signaling (28, 201). Furthermore, GDF15 has been reported to delay gastric emptying at high doses, although this effect is not required for its anorectic actions, as lower doses that induce robust anorexia and pica do not alter gastric emptying rates (201). Notably, the aversive properties of GDF15, including nausea and malaise, do not appear to diminish with prolonged exposure, raising concerns about tolerability in chronic therapeutic settings (201). These considerations are particularly relevant for clinical translation: in a phase 1 trial of the GDF15 receptor agonist LY3463251, dose dependent nausea and emesis were observed in healthy participants, and the resulting weight loss over 12 weeks was modest, suggesting that aversive side effects may limit the therapeutic window of GDF15 based obesity treatments (64). Thus, while GDF15-GFRAL agonism clearly modulates energy balance in humans, the aversive nature of its action represents a significant challenge for clinical weight loss applications.

The GDF15-GFRAL axis has emerged as a highly attractive target for obesity treatment due to its unique mechanism of action, which is largely independent of other appetite regulating hormones such as leptin and GLP-1 (44). Preclinical studies demonstrate that long acting GDF15 analogs and GLP-1/GDF15 dual agonists produce significant weight loss and improve metabolic profiles in DIO mice and non-human primates (202, 203). However, clinical translation has been challenging, as a phase 2 trial of a GDF15 analog showed only modest weight loss in humans with obesity, raising the possibility of blunted GDF15 responsiveness in some individuals, although this observation derives from a single trial and requires confirmation (64). Nevertheless, given its adaptive role in preserving metabolic integrity, strategies to overcome GDF15 resistance, such as combination with GLP-1 receptor agonists, hold promise for future obesity pharmacotherapy (30, 204).

#### 5.1.1 GDF15 in adipose tissue browning and non-shivering thermogenesis

Beyond its appetite suppressing effects through the GFRAL-RET axis, GDF15 has emerged as a significant regulator of energy expenditure via adipose tissue browning and non-shivering thermogenesis. Brown adipose tissue (BAT) itself is a source of GDF15: thermogenic activation of BAT through cold exposure or β3-adrenergic stimulation induces GDF15 expression and secretion from brown adipocytes via protein kinase A mediated mechanisms (205). Single-nucleus RNA sequencing of BAT from cold exposed *Ucp1* knockout mice has revealed a distinct brown adipocyte subpopulation (U2) that exhibits a secretory profile enriched in GDF15, suggesting a shift toward an endocrine role when UCP1 dependent thermogenesis is compromised. Functional analyses demonstrate that GDF15-GFRAL signaling is required to sustain energy expenditure in adipose tissue, and *Ucp1* and *Gfral* double knockout increases food intake to compensate for decreased energy expenditure. A conserved UCP1-GDF15 regulatory axis has been observed in human adipose tissue, suggesting evolutionary conservation of this mechanism (206).

Genetic studies have further established the physiological importance of GDF15 in thermogenesis and energy balance. Mice with selective deletion of *Gdf15* in thermogenic adipocytes exhibit sex-dependent metabolic phenotypes: female knockout mice are resistant to diet induced obesity, associated with increased energy expenditure and improved mitochondrial fatty acid oxidation in BAT, whereas male knockout mice show increased body weight and adiposity upon high fat diet feeding with reduced respiratory capacity in BAT mitochondria. These findings suggest that GDF15 induction in BAT is required to regulate weight gain in a sex dependent manner, potentially through mechanisms independent of GFRAL signaling (207). In the context of mitochondrial stress, BAT derived GDF15 causally contributes to resistance to diet induced obesity and is required for improved cold induced thermogenesis and glucose homeostasis in mice lacking the mitochondrial fusion protein OPA1 (optic atrophy protein 1) in brown adipocytes (208, 209). Furthermore, FGF21 and GDF15 act synergistically to maintain glucose homeostasis and promote resistance to diet induced obesity in mice lacking OPA1 in BAT, highlighting the potential of combined therapeutic strategies (210). In mice with compromised muscle mitochondrial oxidative phosphorylation capacity via respiratory uncoupling (*Ucp1* transgenic mice), GDF15 is induced as a myokine with diurnal variation and is responsible for increased metabolic flexibility, insulin sensitivity, and white adipose tissue browning. Genetic loss of GDF15 in these mice abolishes systemic metabolic flexibility and WAT browning while promoting progressive fat mass gain (38).

Pharmacological evidence further supports GDF15’s role in adipocyte thermogenesis. Berberine, a compound derived from Coptis chinensis, lowers body weight by inducing GDF15 secretion through activation of the ISR in brown adipocytes. The weight change in berberine treated mice is negatively correlated with serum GDF15 levels, and berberine upregulates *Gdf15* mRNA expression and secretion in primary mouse brown adipocytes (211). Additionally, engineered CircRNA-GDF15@LNP formulations that enable sustained GDF15 overexpression activate PPARα/γ signaling and UCP1 expression in liver and adipose tissue to regulate lipid metabolism and adaptive thermogenesis in diet induced obese mice (212). Conversely, chronic exposure to palmitic acid induces brown adipocyte hypertrophy and alters batokine gene expression patterns, including GDF15, suggesting that lipid overload may disrupt the thermogenic and endocrine functions of brown adipocytes (213).

Collectively, these findings position GDF15 as a key regulator of non-shivering adipocyte thermogenesis, complementing its anorexigenic actions to preserve metabolic integrity during caloric excess and mitochondrial stress. However, the relative contributions of GDF15 dependent thermogenesis and appetite suppression to overall energy balance remain to be fully delineated. Furthermore, the mechanisms underlying its thermogenic effects, including whether these are mediated through GFRAL-dependent central pathways, GFRAL-independent peripheral actions, or both, require further investigation.

### 5.2 MASLD

As discussed in Section 4.3, *Gdf15* expression is robustly upregulated in response to metabolic stressors associated with MASLD, including HFD feeding, ER stress, and fatty acid overload (67, 214). In patients with biopsy proven MASLD, circulating GDF15 levels are significantly elevated and correlate positively with disease severity and fibrosis stage (observational association) (215, 216). Additionally, saturated FFAs such as palmitate increase GDF15 expression in Kupffer cells and macrophages, further contributing to circulating GDF15 levels in fatty liver disease (196, 217).

Once elevated, GDF15 exerts protective effects against MASLD progression through multiple mechanisms. Overexpression of *Gdf15* in transgenic mice significantly alleviated HFD-induced hepatic steatosis, improved lipid homeostasis, and enhanced fatty acid β-oxidation without reducing food intake (genetic gain-of-function) (214, 218). Mechanistically, GDF15 protects against hepatic steatosis by inhibiting oxidative stress mediated mitochondrial damage and double strand DNA release, thereby suppressing AIM2 inflammasome activation and the subsequent secretion of IL-18 and IL-1β (214, 218). Furthermore, GDF15 directly suppresses the expression of fibrosis related genes and osteopontin (OPN) in hepatic stellate cells, attenuating liver fibrosis in mouse models of metabolic dysfunction associated steatohepatitis (MASH) (214). GDF15 also induces FGF21 expression in hepatocytes through eIF2α phosphorylation and XBP1 splicing, establishing a GDF15-FGF21 crosstalk that further ameliorates hepatic steatosis and insulin resistance (219). These findings indicate that endogenous GDF15 serves as a compensatory mechanism to limit MASLD progression. In humans, circulating GDF15 levels correlate with MASLD severity and fibrosis stage, but whether this reflects a protective response or simply marks underlying disease burden remains unclear.

Circulating GDF15 levels are consistently associated with the full spectrum of MASLD, from simple steatosis to MASH and advanced fibrosis, making it a valuable non-invasive biomarker for disease staging and risk stratification (216, 217). In a large multicenter study, total and H-specific GDF15 levels were significantly higher in patients with MASH and severe fibrosis compared to controls, and GDF15 showed a strong positive association with the likelihood of having MASLD (215). Moreover, baseline GDF15 levels outperformed other circulating protein biomarkers for the early prediction of MASH occurrence up to 10 years later, with an AUC of 0.90 at 5 years and 0.86 at 10 years (220), however, these findings require validation in independent cohorts before clinical application.

### 5.3 Diabetes

GDF15 expression is robustly upregulated in response to hyperglycemia and metabolic stress associated with diabetes mellitus. In patients with type 2 diabetes, circulating GDF15 levels are significantly elevated compared to healthy controls, and this elevation correlates positively with hemoglobin A1c (HbA1c), fasting glucose, and insulin resistance (observational association) (221). Mechanistically, high glucose induces GDF15 expression in endothelial cells through a ROS- and p53-dependent pathway, while saturated fatty acids such as palmitate upregulate GDF15 via the PERK/eIF2α/CHOP signaling axis in macrophages and hepatocytes (196, 222). In type 1 diabetes (T1D), GDF15 is also elevated and has been shown to be depleted in pancreatic islets under inflammatory conditions, where its translation is blocked by stress granules despite increased circulating levels (48, 223).

Once elevated, GDF15 exerts multiple effects on glucose metabolism and β-cell function. In obese rodents, GDF15 administration improves glycemic control through GFRAL-dependent suppression of food intake and, notably, by enhancing energy expenditure in skeletal muscle via a GFRAL-β-adrenergic signaling axis (40). GDF15 also increases insulin action in the liver and adipose tissue through β-adrenergic receptor-mediated mechanisms, improving insulin sensitivity independently of weight loss (199). In pancreatic β cells, GDF15 plays a critical role in insulin secretion, as *Gdf15* silencing reduces glucose stimulated insulin secretion and increases apoptosis, while GDF15 overexpression protects against cytokine- and palmitate-induced β cell dysfunction (224, 225). This protective effect is mediated through ERBB2 dependent signaling, representing a bona fide GFRAL-independent pathway (Sarkar et al., 2023; PMID: 38076918). However, GDF15 can also promote ER stress induced β cell apoptosis under certain conditions, and *Gdf15* deletion attenuates streptozotocin-induced diabetes by preserving β cell mass (68). These opposing outcomes suggest that the net effect of GDF15 on β-cell fate is context-dependent, likely influenced by the duration and magnitude of GDF15 elevation as well as the underlying metabolic milieu.

Circulating GDF15 levels are consistently associated with diabetes, making it a valuable biomarker for risk stratification and disease monitoring. In a large meta-analysis, higher GDF15 levels were associated with a 2.53-fold increased odds of diabetes, with a dose response relationship showing a 63% increase in diabetes prevalence per 1 ng/mL increase in GDF15 (226). GDF15 also predicts diabetic complications, including diabetic kidney disease, diabetic neuropathy, and diabetic retinopathy, and has been suggested in some studies to offer advantages over traditional biomarkers such as HbA1c for certain outcomes (227–229). However, the interpretation of GDF15 levels in diabetes is modified by factors including disease duration, glycemic control, and the presence of complications such as diabetic kidney disease. Furthermore, the relationship between GDF15 and β-cell function appears to be bidirectional and context-dependent, as discussed above, underscoring that the acute and chronic distinction alone is insufficient to capture the full complexity of GDF15 biology in diabetes.

### 5.4 Cardiovascular disease

As detailed in Section 4.4, GDF15 expression is rapidly and markedly upregulated in response to a wide array of cardiovascular stressors, including myocardial ischemia, pressure overload, and mechanical stretch, as well as in macrophages, endothelial cells, and vascular smooth muscle cells exposed to oxidized LDL or pro-inflammatory cytokines (*in vitro* evidence) (24, 44, 170, 230, 231). Importantly, circulating GDF15 levels increase with age and are further elevated in the presence of cardiovascular risk factors such as hypertension, diabetes, and chronic kidney disease (observational association) (232, 233). These findings are consistent with the view of GDF15 as an integrative readout of cumulative cardiovascular burden, although formal demonstration of its role as a functional sensor awaits mechanistic studies.

The functional role of GDF15 in cardiovascular disease is often paradoxical. In acute settings, rodent models of myocardial infarction and ischemia-reperfusion injury have demonstrated that GDF15 exerts potent cardioprotective effects, limiting infarct size, reducing cardiomyocyte apoptosis, and attenuating injury (animal intervention studies) (24, 122). GDF15 also inhibits neutrophil recruitment and integrin activation, thereby mitigating excessive inflammatory responses that can lead to cardiac rupture following myocardial infarction (genetic evidence) (234). Conversely, in human cohorts, chronically elevated GDF15 levels are consistently associated with adverse outcomes, including heart failure progression, cachexia, and increased mortality (observational association) (235–237). While these human data are observational and cannot establish causality, mouse studies have demonstrated that GDF15 antagonism prevents cachexia and slows heart failure progression, suggesting a contributory role that warrants further investigation. Furthermore, GDF15 deficiency in experimental models has been shown to reduce atherosclerotic lesion formation and attenuate inflammatory responses, suggesting that GDF15 may also promote vascular pathology under certain conditions (238, 239). Importantly, while circulating GDF15 levels consistently predict adverse cardiovascular outcomes in human cohorts, these data are correlational and do not establish that GDF15 itself drives pathogenesis. Elevated GDF15 in patients with worse outcomes may alternatively represent an adaptive stress response mounted by the body to limit disease progression, rather than a direct pathogenic contributor. This distinction is critical, as GDF15 is a stress inducible cytokine whose levels would be expected to rise with greater disease burden regardless of its causal role. Thus, the prognostic value of GDF15 in cardiovascular disease should be interpreted with caution, and causal relationships can only be established through mechanistic studies and randomized intervention trials. This duality underscores that the pathophysiological impact of GDF15 is highly dependent on the duration, magnitude, and cellular context of its elevation.

Elevated circulating GDF15 levels consistently predict adverse cardiovascular outcomes across the entire disease spectrum, from community dwelling individuals to patients with acute coronary syndromes, heart failure, and atrial fibrillation (240–242). These human prognostic data are robust and well-replicated, but they are correlational and do not demonstrate that GDF15 itself drives adverse outcomes. Mechanistic evidence from mouse models suggests that GDF15 can be either protective or deleterious depending on the context, as discussed above. In large cohort studies, GDF15 provides incremental prognostic value beyond traditional risk factors and established biomarkers such as NT-proBNP and high-sensitivity troponin, particularly for predicting cardiovascular death and heart failure hospitalization (243–245). GDF15 is also a key component of the validated ABC (Age, Biomarkers, Clinical history) risk scores for stroke, bleeding, and death in atrial fibrillation patients, highlighting its clinical utility in guiding anticoagulation decisions (244, 246, 247). Future clinical implementation of GDF15 as a biomarker and therapeutic target will require assay standardization, establishment of age- and sex-specific reference ranges, and prospective validation of biomarker guided treatment strategies.

In brief, GDF15 elevation is initially compensatory but becomes maladaptive when stress persists. In obesity, MASLD, diabetes, and cardiovascular disease, acute GDF15 supports metabolic resilience by suppressing appetite, reducing steatosis, improving insulin sensitivity, limiting infarct size. However, chronic elevation signals cumulative disease burden and may actively contribute to β cell apoptosis, cardiac cachexia, or vascular pathology. GDF15 is neither inherently beneficial nor harmful. Its interpretation and therapeutic targeting must distinguish acute adaptive signaling from chronic pathological elevation (**Figure 4**).

**Figure 4:**
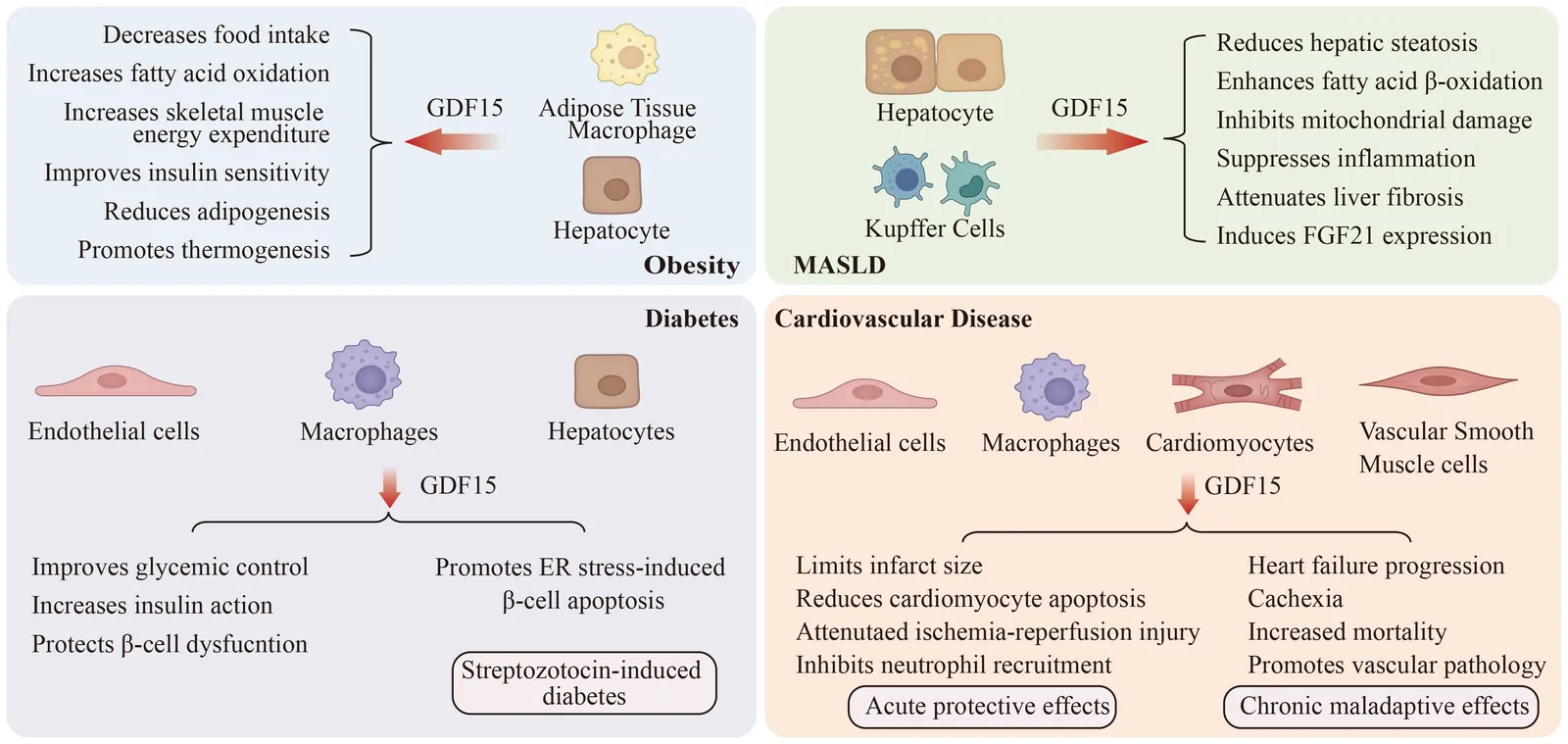
Dual roles of GDF15 in obesity, MASLD, diabetes, and cardiovascular disease. GDF15 exhibits protective and maladaptive effects across obesity, MASLD (metabolic dysfunction-associated steatotic liver disease), diabetes, and cardiovascular disease. In obesity, GDF15 decreases food intake, increases energy expenditure, improves insulin sensitivity, and reduces adipogenesis, collectively counteracting positive energy balance. In MASLD, GDF15 reduces hepatic steatosis, enhances fatty acid β-oxidation, suppresses inflammation, attenuates liver fibrosis, and induces FGF21 (fibroblast growth factor 21) expression in hepatocytes, while also modulating Kupffer cell function. In diabetes, GDF15 improves glycemic control, increases insulin action in peripheral tissues, and protects against β-cell dysfunction. However, under certain conditions, GDF15 can also promote ER stress-induced β-cell apoptosis and exacerbate streptozotocin-induced diabetes, highlighting its dual role in β-cell fate determination. In cardiovascular disease, GDF15 exerts acute protective effects including limiting infarct size, reducing cardiomyocyte apoptosis, attenuating I/R (ischemia-reperfusion) injury, and inhibiting neutrophil recruitment. Conversely, chronic GDF15 elevation is associated with maladaptive outcomes such as heart failure progression, cachexia, increased mortality, and promotion of vascular pathology. This framework emphasizes that GDF15 is neither intrinsically beneficial nor harmful; rather, its impact depends on disease context, duration of elevation, and the balance between compensatory stress signaling and cumulative pathological burden. Furthermore, the biological outcome is modified by host factors including age and metabolic status, which may determine whether GDF15 elevation reflects adaptive compensation or maladaptive pathology. This contextual dependency underscores the need for careful interpretation of GDF15 levels in clinical settings, where these effect modifiers account for a substantial portion of the predictive signal.

## 6 Conclusion and perspectives

Despite significant advances in understanding GDF15 as a stress responsive cytokine, several critical limitations remain in the current literature. First, although multiple tissue sources of GDF15 have been identified, including liver, AT (adipose tissue), skeletal muscle, kidney, intestine, and immune cells (20, 24, 93, 98, 127, 128, 218, 248), the relative contribution of each tissue to circulating GDF15 levels under different disease states remains poorly defined. The lack of systematic tissue specific knockout or tracing models limits our ability to determine which source dominates in which pathological context. Second, while the GFRAL-dependent appetite suppressing pathway in the brainstem has been extensively studied (12–14), non-GFRAL-dependent mechanisms in peripheral tissues are gradually being elucidated. Emerging evidence has identified several alternative receptors or signaling partners, including CD48 in T cells, TGFBR1 and TGFBR2 in macrophages and dendritic cells, ERBB2/HER2 in pancreatic β-cells and cancer cells, and BMPR1A/BMPR2 in trophoblast cells (46–51, 249). However, for other tissues such as vascular endothelium and hypothalamic neurons, the receptor mediating GDF15 effects remains unidentified. This represents a high-priority area for future investigation, as understanding these pathways would enable tissue specific therapeutic modulation of GDF15’s protective or pathological actions without disrupting its central appetite regulating functions. It’s still not completely clear whether additional receptors or direct intracellular actions exist. Third, the dual role of GDF15 as both a protective mediator in acute stress and a maladaptive driver in chronic disease represents a major conceptual gap (22–25). The molecular switch that determines whether GDF15 signals adaptation or pathology has not been identified. A critical limitation of the current literature, which must be emphasized in interpreting clinical associations, is that the vast majority of human studies are correlational. Elevated GDF15 levels in patients with worse outcomes do not necessarily imply that GDF15 is a pathogenic driver of disease progression; rather, they may reflect a compensatory or adaptive response to underlying tissue stress, consistent with the sentinel framework we propose. This distinction is particularly important in cardiovascular disease, where GDF15 elevation could represent an endogenous protective mechanism rather than a maladaptive mediator. Future studies employing Mendelian randomization, longitudinal sampling with dynamic modeling, and randomized interventional trials will be essential to dissect causality from correlation. Until such evidence is available, clinical interpretation of GDF15 levels should remain cautious, and therapeutic strategies targeting GDF15 must be guided by mechanistic understanding rather than associative data alone. We propose that this switch should be defined by measurable parameters rather than vague descriptive terms. Candidate parameters include absolute concentration thresholds such as disease-specific cutoffs established for heart failure or other conditions, temporal dynamics including the rate of change, the duration of elevation, and the cumulative exposure over time, the dominant tissue source of circulating GDF15, and the concurrent activity of other stress pathways such as the integrated stress response, inflammation, or mitochondrial dysfunction. We acknowledge that these parameters remain to be validated, and we present this framework as a hypothesis to guide future research rather than as an established conclusion. Fourth, clinical translation has been slow. Early-phase trials of GDF15 analogs have shown only modest weight loss, which may indicate blunted responsiveness in some populations; however, this finding comes from a single phase 2 trial and should be interpreted cautiously pending replication in larger studies (64). Standardized assays, population specific reference ranges, and biomarker guided intervention studies are currently lacking.

In summary, GDF15 is neither an intrinsically beneficial nor harmful molecule but rather a highly context-dependent sentinel of metabolic integrity. A critical limitation of the current literature is the heterogeneity of evidence: strong causal mechanisms have been established in mouse models, but human data remain predominantly correlational. Moreover, the adaptive versus maladaptive outcome of GDF15 elevation is modified by age, metabolic status, and disease stage, factors that are often overlooked in studies that apply a blunt acute/chronic distinction. Future research should prioritize longitudinal human studies with serial measurements, tissue specific analyses, and stratification by key modifiers to move beyond correlational descriptions toward a mechanistically grounded understanding of GDF15 biology. Its elevation across obesity, diabetes, MASLD, HF, and aging reflects an imbalance between compensatory stress responses and cumulative disease burden. The central task for the next decade is to move from descriptive associations to mechanistic understanding, and ultimately to context-aware interventions that precisely modulate GDF15 signaling according to disease stage, tissue origin, and individual host characteristics to restore metabolic homeostasis.
